# CMOMO: a deep multi-objective optimization framework for constrained molecular multi-property optimization

**DOI:** 10.1093/bib/bbaf335

**Published:** 2025-07-10

**Authors:** Xin Xia, Yajie Zhang, Xiangxiang Zeng, Xingyi Zhang, Chunhou Zheng, Yansen Su

**Affiliations:** The Key Laboratory of Intelligent Computing and Signal Processing of Ministry of Education, School of Artificial Intelligence, Anhui University, Jiulong Road, Hefei 230601, China; The Key Laboratory of Intelligent Computing and Signal Processing of Ministry of Education, School of Computer Science and Technology, Anhui University, Jiulong Road, Hefei 230601, China; College of Computer Science and Electronic Engineering, Hunan University, Lushan Road, Changsha 410012, China; The Key Laboratory of Intelligent Computing and Signal Processing of Ministry of Education, School of Computer Science and Technology, Anhui University, Jiulong Road, Hefei 230601, China; The Key Laboratory of Intelligent Computing and Signal Processing of Ministry of Education, School of Artificial Intelligence, Anhui University, Jiulong Road, Hefei 230601, China; The Key Laboratory of Intelligent Computing and Signal Processing of Ministry of Education, School of Artificial Intelligence, Anhui University, Jiulong Road, Hefei 230601, China

**Keywords:** molecular optimization, constrained multi-objective optimization, deep evolutionary algorithms, dynamic cooperative optimization

## Abstract

Molecular optimization, aiming to identify molecules with improved properties from a huge chemical search space, is a critical step in drug development. This task is challenging due to the need to optimize multiple properties while adhering to stringent drug-like criteria. Recently, numerous effective artificial intelligence methods have been proposed for molecular optimization. However, most of them neglect the constraints in molecular optimization, thereby limiting the development of high-quality molecules that simultaneously satisfy property objectives and constraint compliance. To address this issue, we proposed a deep multi-objective optimization framework, termed CMOMO, for constrained molecular multi-property optimization. The proposed CMOMO divides the optimization process into two stages, which enables it to use a dynamic constraint handling strategy to balance multi-property optimization and constraint satisfaction. Besides, a latent vector fragmentation based evolutionary reproduction strategy is designed to generate promising molecules effectively. Experimental results on two benchmark tasks show that the proposed CMOMO outperforms five state-of-the-art methods to obtain more successfully optimized molecules with multiple desired properties and satisfying drug-like constraints. Moreover, the superiority of CMOMO is verified on two practical tasks, including a potential protein-ligand optimization task of 4LDE protein, which is the structure of $\beta $2-adrenoceptor GPCR receptor, and a potential inhibitor optimization task of glycogen synthase kinase-3$\beta $ target (GSK3$\beta $). Notably, CMOMO demonstrates a two-fold improvement in success rate for the GSK3$\beta $ optimization task, successfully identifying molecules with favorable bioactivity, drug-likeness, synthetic accessibility, and adherence to structural constraints.

## Introduction

Molecular optimization, which aims to improve molecular properties by modifying molecular structures, is a critical step for several engineering applications, such as drug discovery [1] and material science [2]. Molecular optimization presents a fundamental challenge in drug discovery, as it inherently requires the simultaneous optimization of multiple properties that may conflict with each other [3, 4]. Additionally, the practical optimization of molecules often necessitates the adherence to stringent drug-like criteria, thereby preventing some molecules from becoming drug candidates [5, 6]. For instance, in order to discover potent inhibitors against the discoidin domain receptor 1, Zhavoronkov et al. [7] adhered to stringent drug-like criteria, such as avoiding molecules with structural alerts or reactive groups, to select candidates for subsequent synthesis. It should be noted that certain stringent drug-like criteria in molecular optimization are generally not suitable to be treated as optimization objectives. Rather, these criteria are more appropriately implemented as constraints to direct the optimization process. For example, the molecules with either small rings ($<5$ atoms) or large rings ($>6$ atoms) are difficult to synthesize. Although some molecular optimization tasks consider the ring size in optimization objectives, e.g. the penalized logP (PlogP) [8], it is still difficult to rule out the molecules with small or large rings. Consequently, the criterion for molecules with precisely $5$ or $6$ atoms in their rings is typically treated as a constraint in molecular optimization [9, 10]. Thus, the optimization of a specific molecule necessitates the enhancement of multiple properties while simultaneously adhering to several drug-like criteria, that is, the balance between property optimization and constraint satisfaction is required.

Recently, molecular optimization has witnessed significant advancements by the application of artificial intelligence methods [11, 12], such as evolutionary algorithms (EAs) [13, 14], reinforcement learning [15, 16], and gradient-based generative models [17–19]. Most of these existing molecular optimization methods are proposed to enhance a specific property, such as the quantitative estimate of drug-likeness (QED) or the PlogP value. Although these single-property optimization methods perform well in exploring vast chemical search spaces and reducing costs [20], they still face challenges when applied to practical molecular optimization tasks that involve simultaneously enhancing multiple conflicting properties [21, 22]. Subsequently, numerous multi-property optimization methods have been developed to address the simultaneous improvement of multiple molecular properties [23]. For example, QMO [24] and Molfinder [25], which aggregated multiple molecular properties into a single objective for molecule optimization, have exhibited promising performance in the simultaneous optimization of multiple properties. However, the performance of these methods is prone to be affected by the improper setting of weights. Our previous work MOMO [26] employed a multi-objective optimization strategy and identified a set of molecules to enhance the likelihood of successful multi-property optimization of molecules. However, MOMO does not consider the drug-like constraints in the optimization process.

Despite significant progress in multi-property molecular optimization, substantial challenges persist in practical molecular optimization problems, as existing methods often generate molecules that violate essential drug-like constraints. To date, only a few molecular optimization methods have been proposed to deal with constraints by very simple strategies. For example, MSO [27] aggregates all the properties to be optimized and the predefined constraints into a single fitness function, which encounters the difficulty in terms of parameter tuning. GB-GA-P [28] is a genetic algorithm-based method for multiple property optimization, which uses a relatively rough strategy to adhere to drug-like criteria by discarding infeasible molecules. Although the above works generate some molecules that satisfy the constraints, the quality (e.g. the molecular properties) of the resulting molecules still needs to be improved due to the lack of a good balance between the property optimization and the constraint satisfaction.

**Figure 1:**
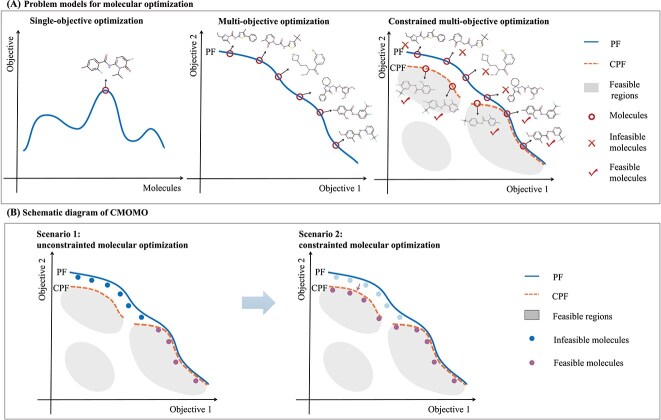
Three problem models for molecular optimization and the schematic diagram of CMOMO. (A) Single-objective optimization aims to find a molecule with the best objective value. Multi-objective optimization aims to search for a set of trade-off molecules among multiple properties in the PF. Constrained multi-objective optimization aims to identify a set of molecules in the CPF that trade off multiple properties and meet drug-like constraints. (B) The optimization process of CMOMO framework. CMOMO firstly focuses on property optimization to find molecules positioned on the PF, and then discovers the molecules on the CPF to solve the CMOMO problem.

Given the importance of balancing property optimization and constraint satisfaction, multiple property molecular optimization task with constraints is suitably modeled as a constrained multi-objective optimization problem. This formulation differs from both single-objective optimization (i.e. the optimization of a single property or the aggregation value of multiple properties) and unconstrained multi-objective optimization. Specifically, the single-objective optimization always discovers a single molecule with the best objective value, and the multi-objective optimization can find a set of molecules with trade-offs among multiple molecular properties (Fig. 1A). Compared to the two aforementioned problems, constrained multi-objective molecular optimization CMOMO is more complex, since it explores molecules that not only compromise different molecular properties, but also satisfy some predefined drug-like constraints. What’s more, these constraints may result in the narrowness, disconnection and irregularity of feasible molecular space (Fig. 1A). Therefore, it is challenging to explore feasible molecules (i.e. the molecules adhere to constraints) with multiple desired properties.

To address the above issues, in this study, we propose a CMOMO framework to simultaneously optimize multiple molecular properties while satisfying several constraints. CMOMO first solves the unconstrained multi-objective molecular optimization scenario to find molecules with good properties, and then considers both properties and constraints to find feasible molecules that possess promising properties (Fig. 1B). The main contributions of this paper are summarized as follows:

- A CMOMO framework is proposed to address the multi-property molecular optimization with several drug-like constraints. CMOMO achieves a good balance between the optimization of multiple properties and the satisfaction of constrained molecules by first searching in the unconstrained scenario and then identifying feasible molecules with the desired property values in the constrained scenario. Besides, a latent vector fragmentation based evolutionary reproduction VFER strategy is designed to optimize molecules effectively. To the best of our knowledge, CMOMO is the first method that delicately balances property optimization and constraint satisfaction for molecular optimization, and thereby yielding high-quality molecules exhibiting desired molecular properties while adhering rigorously to drug-like constraints.
- CMOMO facilitates the simultaneous optimization of multiple molecular properties while adhering to various drug-like constraints, and subsequently identifies a set of optimal molecules with trade-offs among multiple objectives. We demonstrated the high performance of CMOMO in a variety of molecular optimization tasks. Via CMOMO, we identified a collection of potential ligands of $\beta $2-adrenoceptor GPCR receptor (4LDE) and potential inhibitors against glycogen synthase kinase-3 target (gsk3$\beta $) with multiple higher properties while adhering to the drug-like constraints.

## Materials and methods

### Constrained molecular multi-property optimization formulation

In constrained multi-property molecular optimization problems, each property to be optimized is treated as an optimization objective, and the strict requirements are treated as constraints. The problems can be mathematically expressed as follows. 


(1) \begin{align*}& \begin{array}{ll}\text{ Maximize} & \mathbf{F}(x)=\left(f_{1}(x), \cdots, f_{m}(x)\right), \\ \text{ subject to } & x \in \Omega \\ & g_{i}(x) \leq 0, \quad i=1, \cdots, p, \\ & h_{j}(x)=0, \quad j=1, \cdots, q.\end{array}\end{align*}

where $x$ represents a molecule and $\Omega $ represents the molecular search space. $\mathbf{F}(x)$ is the objective vector consisting of $m$ optimization properties, i.e. $f_{1}(x), \cdots , f_{m}(x)$. The $g_{i}(x)$ and $h_{j}(x)$ are the $i$th inequality constraint and the $j$th equality constraint, respectively.

The constraint violation (*CV*) aggregation function to measure the CV degree of a molecule [29, 30], which is defined as follows. 


(2) \begin{align*}& CV(x)=\sum_{i=1}^{p} \text{max}{(g_{i}(x),0)} + \sum_{j=1}^{q} |h_{j}(x)|.\end{align*}

If $CV(x)$ is equal to zero, the molecule $x$ is said to be feasible; otherwise, it is an infeasible molecule.

As can be seen from Equation (1), the problem is flexible and scalable, where the optimization objectives can be comprehensive evaluation metrics, non-biological active properties, and biological active properties [31]. As for the constraints, they can be the constraints concerning the ring size, substructure constraints, skeleton constraints [32, 33], and many others. It is necessary to transform the specific constraint into an equality or inequality constraint to compute the CV degree.

### The proposed CMOMO framework

In this study, each property to be optimized is treated as an optimization objective and the stringent drug-like criteria are treated as constraints. To this end, a tailored dynamic constraint handling strategy divides the optimization into two scenarios (unconstrained scenario and constrained scenario) and dynamically deals with the constraints in these two scenarios, thereby achieving a dynamic balance between property optimization and constraint satisfaction. Furthermore, a vector fragmentation-based evolutionary reproduction strategy (VFER) significantly enhances the efficiency of evolution in the continuous implicit space. The above two strategies within the dynamic cooperative optimization help to enhance the ability to identify molecules that possess desirable molecular properties while strictly adhering to drug-like constraints. The procedure of the proposed CMOMO is illustrated as follows.

**Figure 2:**
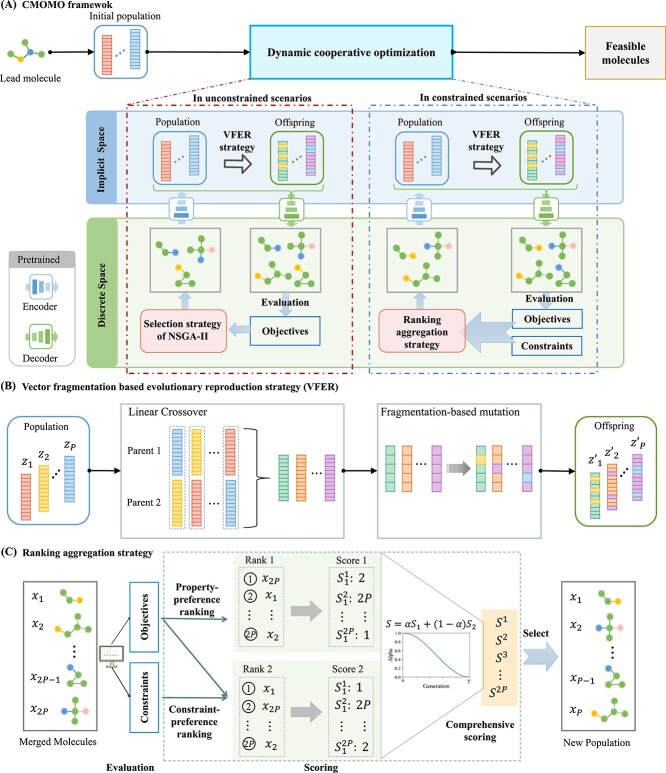
The illustrative diagram of CMOMO. (A) To begin with, CMOMO generates an initial population with $P$ molecules for a lead molecule. Then, CMOMO performs the dynamic cooperative optimization. Finally, CMOMO achieves a set of feasible molecules (with desired molecular properties and under the drug-like constraints). (B) The VFER strategy. The VFER strategy is employed to generate promising offspring molecules through linear crossover and fragmentation-based mutation operations. (C) The ranking aggregation strategy. This strategy dynamically aggregates the rankings of molecules that ordered by properties and constraints, respectively.

(1) Population initialization: Given a lead molecule represented by a SMILES string, CMOMO first utilizes existing public database to construct a Bank library, which contains high property molecules that are similar to the lead molecule. Then, CMOMO uses a pre-trained encoder [34] to embed the lead molecule and the molecules in the Bank library into a continuous implicit space. It is worth noting that the pre-trained encoder-decoder constructs a latent continuous space to facilitate more efficient and smooth exploration, which has been widely used in many existing methods, such as QMO and MOMO [24, 26]. Next, CMOMO performs a linear crossover [35] between the latent vector of lead molecule and that of each molecule in the Bank library, which enables CMOMO to generate a high-quality initial molecular population.
(2) Dynamic cooperative optimization: In this step, the cooperative optimization between the discrete chemical space and the continuous implicit space is dynamically executed in both unconstrained and constrained scenarios. To be specific, in the unconstrained scenario, CMOMO first employs a newly designed VFER strategy (VFER) on the implicit molecular population to efficiently generate offspring molecules in the continuous implicit space (Fig. 2B). Next, CMOMO decodes parent and offspring molecules by the pre-trained decoder from the continuous implicit space to the discrete chemical space to evaluate their molecular properties [34]. It is worth noting that only a few invalid molecules are generated from the decoded offspring (see Supplementary Information S1), which are filtered out by RDKit based validity verification before population update. Further, the molecules with better property values are selected by the environmental selection strategy of NSGA-II [36] to obtain the next molecular population.

CMOMO iteratively performs the above operations until reaching the given number of iterations, which enables CMOMO to possess strong ability to explore the vast search space to obtain molecules with good convergence and diversity, where convergence measures the proximity of molecules to the constrained Pareto front (CPF) and diversity assesses the distribution of molecules in the search space. Afterwards, CMOMO switches to the optimization in the constrained scenario. In comparison to the optimization in the unconstrained scenario, the optimization in the constrained scenario adopts a novel way (i.e. a ranking aggregation strategy) to evaluate and select molecules by balancing property optimization and constraint satisfaction (Fig. 2C). Finally, we identify feasible molecules that have excellent molecular properties and satisfy the drug-like constraints.

### Population initialization strategy

In the proposed CMOMO, the population initialization strategy is used to generate a set of high-property molecules that are similar to the lead molecule. To this aim, we first build a Bank library for each lead molecule by screening high-property molecules from public databases [37–39] (Supplementary Fig. S1A). Then, we use the pre-trained encoder to encode the lead molecule and screened molecules which are represented by SMILES strings into the continuous implicit space. Next, a linear crossover operation is performed between the latent vector of lead molecule and that of each screened molecule to obtain a set of new latent vectors (Supplementary Fig. S1B). Finally, these newly generated latent vectors are decoded from the continuous implicit space to the discrete chemical space. In this way, molecules in the initial population possess not only the genes of lead molecule but also those of screened molecules, which enables initial molecules to have good properties under the premise that they are similar to the lead molecule.

### Molecule generation strategy

In the two optimization scenarios, CMOMO utilizes pre-trained encoder and decoder to map molecules between the discrete chemical space and continuous implicit space, which enables it to generate molecules effectively as QMO [24] and MSO [27]. Besides, a VFER strategy is designed in CMOMO to further enhance the effectiveness and efficiency of generating offspring molecules, which consists of two operations, i.e. blended linear crossover and fragmentation-based mutation. To be specific, CMOMO first selects two latent vectors randomly as parent molecules to perform linear crossover, then, the generated vectors are divided into small fragments with one fragment being selected randomly to perform mutation.

#### Crossover

In the process of generating offspring molecules, the crossover operation is mainly used to inherit the good genes of the parent molecules. To this aim, the blended linear crossover operator [35] is used in CMOMO, which has also been widely used by many other methods, including DEL [40] and MOMO [26]. Specifically, given a set of latent vectors of molecules, two vectors (${z_{1}}$ and ${z_{2}}$) are randomly selected as parent molecules to generate two offspring molecules (${{z^{\prime}}_{1}}$ and ${{z^{\prime}}_{2}}$) by the following way. 


(3) \begin{align*}& \left\{ \begin{array}{l} {{z^{\prime}}_{1}} = {z_{1}} + ( - d + (1 + 2d){u_{1}})({z_{2}} - {z_{1}}),\\{{z^{\prime}}_{2}} = {z_{1}} + ( - d + (1 + 2d){u_{2}})({z_{2}} - {z_{1}}), \end{array} \right.\end{align*}

where ${u_{1}}$ and ${u_{2}}$ are two random numbers that distribute uniformly between 0 and 1. The $d \ge 0$ is a parameter that controls whether the search space is interpolated or extrapolated by the blended linear crossover operator.

#### Mutation

**Figure 3:**
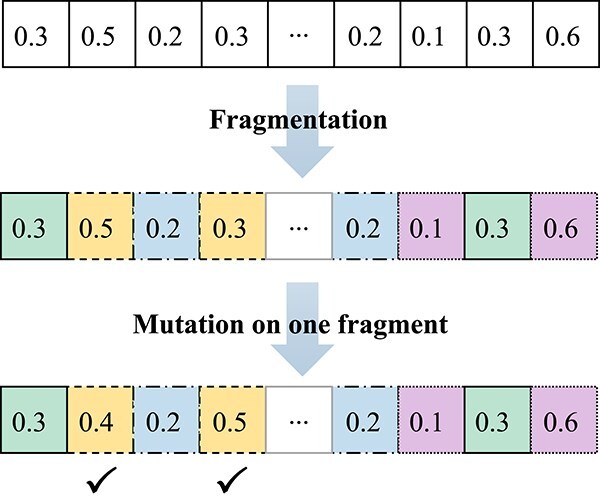
Illustrative example of the proposed fragmentation-based mutation operator.

In the proposed algorithm, the mutation operator is used to introduce some new genes, which prevents molecules from trapping into local optima. Besides, since the length of latent vectors can be very large (e.g. 512 in QMO, MSO and the proposed CMOMO), herein a fragmentation-based mutation operator is designed. As shown in Fig. 3, given a latent vector generated by the crossover operator, the mutation operator first divides it into some small fragments. Then, the mutation operator selects a fragment randomly to mutate all genes in it with the mutation probability ${p_{m}}$. In this way, the dimension of the long latent vector can be reduced considerably, which alleviates the issue of the curse of dimensionality.

### Dynamic constraint handling strategy

When CMOMO adopts the same strategy to generate high-quality molecules in the two scenarios, the two optimization scenarios are different in terms of molecule evaluation and selection. Specifically, while the first scenario evaluates and selects molecules by only considering property values, the second scenario takes both property values and CV degree into consideration. In this way, CMOMO dynamically addresses the complex constraints in multi-property molecular optimization, yielding final results that satisfy all constraints while maintaining good convergence and diversity.

#### Molecule evaluation and selection in the first scenario

In the proposed CMOMO, the first scenario is an unconstrained multi-objective molecular optimization scenario, which aims to find molecules with good convergence and diversity. To this aim, when CMOMO in this scenario evaluates molecules by considering only their property values, the environmental selection strategy of NSGA-II is used to select molecules from the union of parent and offspring molecules, which consists of two components, i.e. non-dominating sorting and crowding distance calculation. The specific steps of NSGA-II can be found in Supplementary Information S3 or the original paper [36].

In the first scenario, when the front number and crowding distance of each molecule are obtained by NSGA-II, the proposed CMOMO selects $P$ molecules from the union of parent and offspring molecules that contains $2P$ molecules, where the front number is used as the first selection criterion and the crowding distance is adopted as the second selection criterion. Specifically, CMOMO first selects molecules front by front until the number of molecules in $F_{1} \bigcup F_{2} \bigcup \cdots \bigcup F_{k}$ is larger than $P$. Then, CMOMO deletes $|F_{1} \bigcup F_{2} \bigcup \cdots \bigcup F_{k}| - P$ molecules with the smallest crowding distance from $F_{k}$. In this way, CMOMO obtains $P$ molecules with good convergence and diversity to undergo further optimization.

#### Molecule evaluation and selection in the second scenario

Different from the first scenario, the second scenario is a CMOMO scenario, which poses high requirement on the balance between property optimization and constraint satisfaction. To address this issue, a ranking aggregation strategy is designed to perform molecule selection when both property values and CVs are considered in the molecule evaluation in the second scenario, which consists of three steps, i.e. property-preference ranking, constraint-preference ranking, and comprehensive scoring (Fig. 2C).

**Property-preference ranking.** The property-preference ranking considers only property values of molecules. Given the union of parent and offspring molecules which contains $2P$ molecules, when their front numbers and crowding distances are obtained by non-dominated sorting and crowding distance, these $2P$ molecules are ranked by the following ways. First, the molecules are ranked based on their front numbers, where the molecules having smaller front number are ranked ahead of that having larger front number. Then, the molecules having the same front number are ranked based on their crowding distances, where the molecules having larger crowding distances are ranked ahead of that having smaller crowding distances. In this way, each molecule in the union of parent and offspring molecules obtains a unique rank number ranging from 1 to $2P$.

**Constraint-preference ranking.** The difference between the constraint-preference ranking and property-preference ranking lies in the fact that the constraint-preference ranking takes both property values and CV degree into consideration. When the two ranking operations rank molecules in the union of parent and offspring molecules according to front numbers and crowding distances, they use different methods to obtain the front numbers of molecules. Specifically, in property-preference ranking, front numbers of molecules are obtained by non-dominated sorting, which does not take constraints into account. By contrast, in constraint-preference ranking, front numbers of molecules are obtained by the constraint dominance principle. Given two molecules ${x_{1}}$ and ${x_{2}}$, the molecule ${x_{1}}$ is said to dominate molecule ${x_{2}}$ if one of the following conditions is met: (i) both ${x_{1}}$ and ${x_{2}}$ are feasible, besides, ${x_{1}}$ has better property values than ${x_{2}}$. (ii) ${x_{1}}$ is feasible while ${x_{2}}$ is infeasible; (iii) both ${x_{1}}$ and ${x_{2}}$ are infeasible, besides, the CV degree of ${x_{1}}$ is smaller than that of ${x_{2}}$.

**Comprehensive scoring.** When the above two ranking operations are performed, each molecule obtains two rank numbers, which are herein used to perform a comprehensive scoring for all molecules. Specifically, the rank numbers obtained in property-preference ranking are saved in $S_{1}$ with $S_{1}^{i}$ representing the first score of the $i$th molecule, while the rank numbers obtained in constraint-preference ranking are saved in $S_{2}$ with $S_{2}^{i}$ representing the second score of the $i$-th molecule. In this way, each molecule in the union of parent and offspring molecules obtains a comprehensive score by aggregating its two scores as follows, 


(4) \begin{align*}& \begin{array}{l} S^{i} = \alpha{S_{1}^{i}} + (1 - \alpha ){S_{2}^{i}},\\ \alpha = \frac{1}{2} \times \left(1 + \cos\left (\frac{t}{T}\pi \right)\right). \end{array}\end{align*}

where the $S^{i}$ represents the comprehensive score of the $i$th molecule, the $\alpha $ is a parameter which gradually decays from 1 to 0 as the evolutionary generation $t$ increases.

Based on the comprehensive score of each molecule, CMOMO selects $P$ molecules with the smallest comprehensive scores from the union which contains $2P$ parent and offspring molecules. Furthermore, CMOMO dynamically adjusts the effects of property-preference ranking and constraint-preference ranking on the comprehensive scoring mechanism. In the early stages, the parameter $\alpha $ is designed to shrink very slowly to prioritize molecular property optimization without considering too many constraints, which enables CMOMO to perform comprehensive global exploration. In the middle stage of the evolution, $\alpha $ decays fast, which facilitates the transition from property priority to constraint priority optimization. Finally, in the later stages, the parameter $\alpha $ enables CMOMO to explore the constrained Pareto front (PF) by emphasizing the constraints. The analysis of the CMOMO with different decay functions can be found in Supplementary Information S4, where it is shown that the CMOMO with cosine-based decay obtains a relatively better performance.

## Results

### Experimental design

To verify the performance of the CMOMO framework, we compare the proposed CMOMO with five state-of-the-art molecular optimization methods on four molecular optimization tasks.

#### Optimization tasks

The optimization objectives and constraints of two benchmark tasks (Task 1 and Task 2) and two practical tasks (Task 3 and Task 4) are described as follows.

**Optimization objectives.** Task 1 aims to simultaneously optimize three non-biological activity properties, including the drug likeness (QED) [41], the improvement of the penalized partition ratio of the solute between octanol and water (PlogP_imp) [42], and the Tanimoto similarity [43] between optimized molecules and lead molecules (Similarity).

Task 2 optimizes three structural scores of a FDA-approved drug (Perindopril) [44] and the Similarity. The three structural scores are the dissimilarity score between optimized molecules and Perindopril (Score_dissim), the molecular weight score of optimized molecules (Score_mw), and the rotatable bond score of optimized molecules (Score_rb).

The optimization objectives of Task 3 are QED, the simulated docking score with the $\beta $2-adrenoceptor GPCR receptor (4LDE), and Similarity. The 4LDE protein is responsible for muscle relaxation and bronchodilation [45], where the docking scores between molecules and 4LDE protein are simulated by autodock [46].

The optimization objectives of Task 4 are QED, the predicted GSK3$\beta $ inhibition to glycogen synthase kinase-3 target, the normalized synthetic accessibility (SA), and Similarity. GSK3$\beta $ is known to be implicated in the pathogenesis of several diseases and is a potential target for Alzheimer’s disease treatment. Since this task is an experimental inhibitory activity optimization task, we followed the settings of existing studies [11, 20, 47] to predict the inhibition of molecules against GSK3$\beta $ target by a pre-trained surrogate model [48]. This model has been integrated into the well-known drug discovery platform TDC as an evaluator [49].

It is worth noting that, in the four tasks, the 4LDE score is the smaller the better, and other properties are the larger the better.

**Constraints.** We consider two fundamental structural constraints in the above four tasks. As highlighted in previous studies, molecules featuring small rings (comprising three or four atoms) often exhibit instability [9], while those containing large rings (with more than 6 atoms) pose significant challenges in synthesis [10]. These ring-related constraints have been considered in existing molecular optimization frameworks, such as GB-GA-P [28], which explicitly excludes molecules with large rings during optimization process. Thus, the first constraint in our experiments (denoted as ${C_{1}}$) is that the rings in molecules should have only five or six atoms, which affects the synthesis of molecules. Given a molecule which contains $K$ rings with each ring having ${r_{k}}$ atoms, its CV on ${C_{1}}$ is calculated as 


(5) \begin{align*}& {\sigma_{1}}(x) = \sum\nolimits_{k = 1}^{K} {[\max \{ {r_{k}} - 6,0\} + \max \{ 5 - {r_{k}},0\} ]}.\end{align*}

As experts have noted, certain substructures are associated with reactivity or toxicity issues, and their presence in a molecule can lead to undesirable properties [50]. Consequently, molecules generated in existing drug design workflows typically undergo a screening process to filter out such toxic substructures [7]. Thus, the second constraint (denoted as ${C_{2}}$) is that molecules cannot contain toxic or uncommon substructures. We adopt a set of 163 substructures compiled in the molecular optimization method as constraints [27]. Specially, given a molecule $x$, the 163 substructures in MSO [27] are considered to calculate the CV ${\sigma _{2}}(x) = \left | \xi \right |$ on ${C_{2}}$, where $\left | \xi \right |$ denotes the number of toxic or uncommon substructures in $x$.

Based on ${\sigma _{1}}(x)$ and ${\sigma _{2}}(x)$, the aggregated CV degree of molecule $x$ can be calculated as follows. 


(6) \begin{align*}& CV(x) = \sum\nolimits_{c = 1}^{2} {\frac{1}{{{{\max} _{{x_{i}} \in \psi}} {\sigma_{c}}({x_{i}})}}{\sigma_{c}}(x)},\end{align*}

where $\psi $ is the set of molecules. The larger the value of $CV$, the larger the degree of CV of the molecule.

#### Datasets

Four datasets are employed in four tasks, each molecule in the data sets is optimized separately as a lead molecule. Dataset 1 for Task 1 is 500 molecules with $QED \in [0.6,0.8]$ selected from the PlogP dataset [8]. The dataset 2 for Task 2 is a random selection of 800 molecules from the publicly available dataset [44]. The lead molecules for Task 3 are 100 molecules selected from the publicly available dataset from Nigam et al. [45] with $\Delta{E_{4LDE}} \in [ - 7.9, - 7]$ and $QED \in [0.7,0.8]$. The dataset used for Task 4 consists of 800 molecules with $QED \in [0.6,0.8]$, $GSK3\beta \in [0,0.5]$, and $SA \in [0.6,0.8]$ from the GSK3$\beta $ dataset [16]. The detailed construction process of Bank library and the selection criteria for the four tasks are provided in Supplementary Information S5.1. The analysis of the impact of different Bank sizes on model performance can be found in Supplementary Information S6.

#### Comparison methods

In the experiments, there are totally five state-of-the-art methods that are selected to compare with the proposed CMOMO, namely QMO [24], Molfinder [25], MOMO [26], MSO [27], and GB-GA-P [28]. In these five comparison methods, QMO, Molfinder, and MOMO deal with constraints by selecting feasible molecules from their final optimization results, MSO treats constraints by aggregating them with optimization objectives, and GB-GA-P addresses constraints by discarding infeasible molecules at each iteration. Table 1 summarizes the comparative analysis of CMOMO against baseline methods across the constraint handling, property handling, optimization approach, and search space. It is worth noting that, in the experiments, all methods use the same population size/number of samples and the same iterations, which enables the performance comparison to be fair. Details about the parameters can be found in the Supplementary Information S5.2.

**Table 1:** Comparative analysis of CMOMO against comparison methods

Methods	Constraint handling strategy	Multi-property handling strategy	Optimization approach	Search space
QMO	Discarding of infeasible molecules in the last generation	Pareto-based	Zeroth-order gradient optimization	Latent space
Molfinder	Discarding of infeasible molecules in the last generation	Aggregation-based	EA	Discrete space
MOMO	Discarding of infeasible molecules in the last generation	Pareto-based	EA	Latent space
MSO	Penalty function	Aggregation-based	EA	Latent space
GB-GA-P	Discarding of infeasible molecules at each generation	Pareto-based	EA	Discrete space
CMOMO	Dynamic constraint handling	Pareto-based	EA	Latent space

#### Evaluation metrics

We compare the performance of the six methods by using four metrics, namely the optimization success rate (SR), the number of successfully optimized molecules (${N_{SR}}$), the hypervolume (HV), and the mean property value of successfully optimized molecules. Specifically, SR is the ratio of successfully optimized molecules against all molecules to be optimized, where successfully optimized molecules refer to those feasible molecules whose property values are better than the predefined thresholds. The thresholds on the four tasks can be found in the Supplementary Information S5.3. The HV [51] is used to comprehensively evaluate both convergence and diversity of successfully optimized molecules, which can be obtained by calculating the size of the hyperspace between the set of molecules and the reference point, where the reference point in each task is set as a zero vector.

### CMOMO outperforms the comparison methods on benchmark tasks

**Figure 4:**
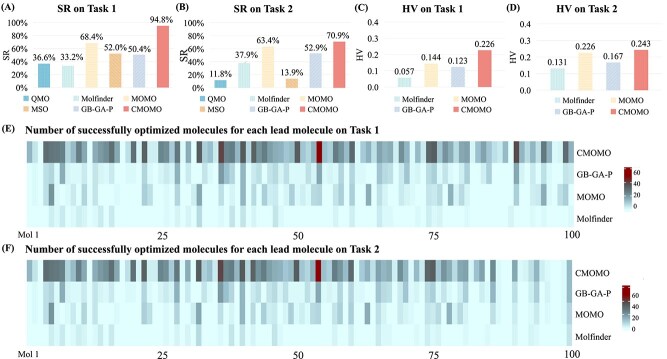
Performance of CMOMO and comparison methods on two benchmark constrained multi-objective optimization tasks. (A) SR of CMOMO and comparison methods on Task 1. (B) SR of CMOMO and comparison methods on Task 2. (C) HV of CMOMO and three Pareto optimization methods on Task 1. (D) HV of CMOMO and three Pareto optimization methods on Task 2. (E) The number of successfully optimized molecules obtained by four multi-objective optimization methods on Task 1. (F) The number of successfully optimized molecules obtained by four multi-objective optimization methods on Task 2.

Figure 4 shows the SR, HV, and the number of successfully optimized molecules by the proposed CMOMO and comparison methods. From the figure, the following three observations can be obtained.

First, the proposed CMOMO achieves the highest SR in comparison with the five molecule optimization methods on the two benchmark tasks (Fig. 4A and Fig. 4B). For example, on Task 1, the proposed CMOMO successfully optimized $94.8\%$ of $800$ molecules, which achieves significantly better performance than all the five comparison methods. It is indicated that the proposed CMOMO is likely to successfully optimize a molecule which is hard to be optimized.

Second, the successfully optimized molecules achieved by the proposed CMOMO have superior molecular properties and exhibit greater diversity, where the diversity can be measured by the number and the distribution of molecules. (i) To comprehensively assess the quality of the successfully optimized molecules, Fig. 4C and D present the HV of the optimized molecules achieved by the proposed CMOMO and other three considered multi-objective methods, i.e. Molfinder, MOMO, and GB-GA-P. As can be seen from the figure, CMOMO obtains the largest mean HV values on the two tasks, which indicates that the proposed CMOMO is capable of generating a wide variety of molecules that have superior molecular properties. (ii) To further access the quality of the successfully optimized molecules, we plotted the mean property values of successfully optimized molecules in Supplementary Fig. S5A and B in the Supplementary Information. The results show that the proposed CMOMO achieves almost the largest property improvement, which indicates that CMOMO can enhance multiple properties of molecules. (iii) To check the diversity of the resulted molecules corresponding to each molecule to be optimized, Fig. 4E and F present the number of successfully optimized molecules obtained by four multi-objective optimization methods (CMOMO, GB-GA-P, MOMO, and Molfinder) for $100$ lead molecules. The successfully optimized molecules for all lead molecules are also given in Fig. S6A and B in the Supplementary Material. We can see from the figure that CMOMO achieved the largest number of successfully optimized molecules for each molecule.

To validate the reliability of the optimization results, we conduct significance analysis to compare the results of compared methods with CMOMO across lead molecules. The results in Supplementary Information S9 show that CMOMO significantly outperforms the comparison methods on Task 1 and Task 2. From the above empirical results, we can conclude that CMOMO is a promising constrained multi-property molecular optimization method. Besides, the way to deal with constraints by balancing them with property optimization is superior to other existing constraint-handling strategies (e.g. merely selecting feasible candidates from the final optimization outcomes or discarding those deemed infeasible). The average running times of CMOMO and the compared methods are shown in Supplementary Information S10, where MSO requires the most computational time and CMOMO is computationally more expensive than the other four methods.

### CMOMO exhibits good performance on finding potential ligands for the 4LDE protein

**Figure 5:**
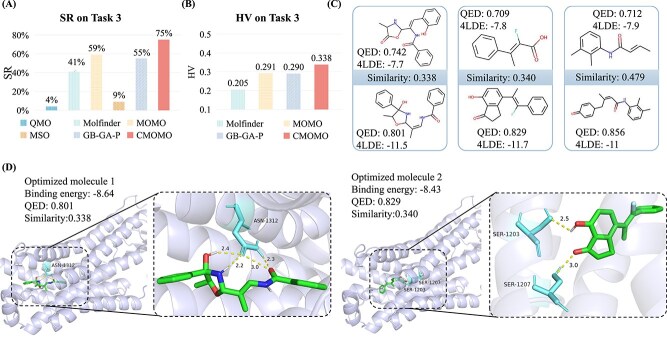
Performance of CMOMO and comparison methods on Task 3. (A) SR of CMOMO and comparison methods on Task 3. (B) HV of CMOMO and three Pareto optimization methods on Task 3. (C) The optimization results obtained by CMOMO on five test instances, where the molecules at the top and bottom rows denote the lead and optimized molecules, respectively. (D) The docking pose, QED, binding energy, and Similarity of two optimized molecules for the 4LDE protein are shown. Both molecules exhibit the desired QED, binding energy, and Similarity. The right two sub-figures show the interactions between the optimized molecules and the amino acid residues in protein binding pockets.

In this subsection, we test the performance of CMOMO and comparison methods on the protein-ligand optimization task. First, we give the results of the six considered methods in terms of SR. As shown in Fig. 5A, the proposed CMOMO obtains the largest SR of 75%, while the largest SR obtained by the comparison methods is 59%, which is obviously smaller than the proposed CMOMO. Then, we present their optimization results in terms of HV of successfully optimized molecules, which are shown in Fig. 5B, the results show that the proposed CMOMO obtains the largest HV value than the other three Pareto optimization methods. Next, we compare the performance of CMOMO and comparison methods by comparing the quality and quantity of their successfully optimized molecules. The results in Fig. S5C show that the molecules obtained by CMOMO have better properties. The results in Fig. S6C show that CMOMO obtains a larger number of successfully optimized molecules than the three Pareto optimization based comparison methods for most lead molecules. The significance analysis on Task 3 demonstrates that CMOMO significantly outperforms the comparison methods (Supplementary Information S9).

To better validate the performance of CMOMO, we provide three pairs of molecules with each pair containing a lead molecule and an optimized molecule to compare their difference. As shown in Fig. 5C, it can be seen that the proposed CMOMO improves their QED and 4LDE considerably; besides, for each pair of molecules, the optimized and lead molecules are similar, which possess a similarity value that is larger than 0.3. Then, the first two molecules shown in Fig. 5C are used to conduct the protein-ligand interaction analysis, where the docking software Autodock and the visualization software PyMol [52] are used to simulate the docking energies and the top docking poses. As shown in Fig. 5D, the binding energies of the two successfully optimized molecules for the 4LDE protein are smaller than −7 kcal/mol, where the −7 kcal/mol has been widely used as a threshold to assess whether a molecule is drug-like in some researches [53, 54]; the low binding energies suggest that these molecules hold potential as ligands for the 4LDE protein. Moreover, the protein-ligand docking poses and the interactions between the two molecules and the 4LDE protein are visualized in Fig. 5D. It can be seen that the two successfully optimized molecules form 4 to 2 hydrophobic interactions (represented by yellow dashed lines) with the amino acid residues in the binding pocket of 4LDE, respectively.

Overall, the above statistical results in terms of four evaluation metrics show that the proposed CMOMO outperforms the comparison methods to find potential 4LDE protein ligands; besides, the protein-ligand interaction analysis results show that the successfully optimized molecules obtained by CMOMO have the potential to bind with the 4LDE protein.

### CMOMO performs well on finding potential inhibitors against the GSK3$\beta $ target

**Figure 6:**
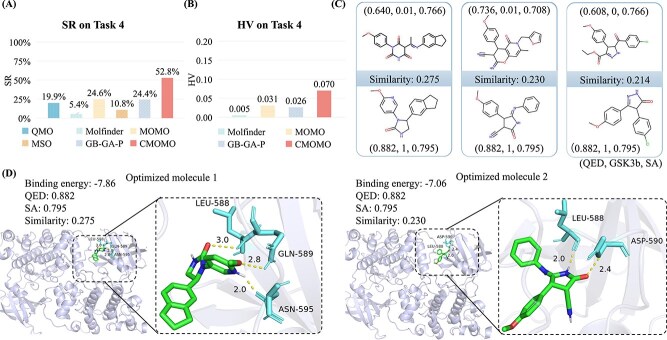
Performance of CMOMO and comparison methods on Task 4. (A) SR of CMOMO and comparison methods on Task 4. (B) HV of CMOMO and three Pareto optimization methods on Task 4. (C) The optimization results obtained by CMOMO on five test instances, where the molecules at the top and bottom rows denote the lead and optimized molecules, respectively. (D) The docking poses, QED, binding energy, SA, and Similarity of two optimized molecules for the GSK3$\beta $ target are shown. Both molecules exhibit the desired QED, binding energy, SA, and Similarity. The right two sub-figures show the interactions between the optimized molecules and the amino acid residues in protein binding pockets.

Figure 6 presents the results on Task 4, i.e. the inhibitor optimization task, which shows that the proposed CMOMO holds the ability to find potential inhibitors against GSK3$\beta $. To be specific, the proposed CMOMO outperforms the comparison methods in terms of four evaluation metrics, including the SR, HV, mean property value, and the number of successfully optimized molecules. Besides, the protein-ligand interaction analysis shows that the molecules obtained by CMOMO have potential inhibitions against the GSK3$\beta $ target.

As shown in Fig. 6A, while the SRs obtained by the five comparison methods range from 5.37% to 24.6%, CMOMO achieves the largest SR of 52.7%, which is obviously larger than that obtained by the comparison methods. In terms of HV, Fig. 6B shows that CMOMO obtains the largest mean hypervolume, which is larger than that obtained by three Pareto optimization methods. As for the quality and quantity of successfully optimized molecules obtained by CMOMO and comparison methods. The results in Supplementary Fig. S5D show that the successfully optimized molecules obtained by CMOMO have the best performance on multiple properties, especially in terms of GSK3$\beta $ inhibition and SA. The results in Supplementary Fig. S6D show that CMOMO obtains a larger number of successfully optimized molecules for most lead molecules than comparison methods. The significance analysis of CMOMO and compared methods can be found in Supplementary Information S9.

To better validate the performance of the proposed CMOMO, we use three successfully optimized molecules to compare with their corresponding lead molecules. As shown in Fig. 6C, for each lead molecule, the proposed CMOMO obtains a successfully optimized molecule with the GSK3$\beta $ inhibition, QED and SA being improved considerably. Moreover, we conduct an in-depth analysis of protein-ligand interactions for the first two optimized molecules in Fig. 6C, where the protein structure of GSK3$\beta $ is downloaded from the UniProt website [55]. As depicted in Fig. 6D, the binding energies of the two optimized molecules are also smaller than −7 kcal/mol, when the two molecules generate 3 and 2 hydrophobic interactions (represented by yellow dashed lines) with the amino acid residues of GSK3$\beta $, respectively. The above results illustrate that the molecules obtained by CMOMO have the potential to bind with the GSK3$\beta $ target.

### Evolutionary process analysis

In this subsection, we randomly select a lead molecule in Task 1 to investigate the search capability of the proposed CMOMO and the evolutionary trajectory of optimized molecules. Supplementary Fig. S7 gives the optimization results obtained by the proposed CMOMO and the three Pareto optimization based comparison methods, where each dot represents a successfully optimized molecule with its size reflecting the similarity to the lead molecule. It can be seen that the number of successfully optimized molecules obtained by CMOMO is obviously larger than that obtained by the three comparison methods. Besides, the successfully optimized molecules obtained by CMOMO dominate the ones obtained by the comparison methods, which means that the molecules obtained by CMOMO have better QED, PlogP, and Similarity properties.

**Figure 7:**
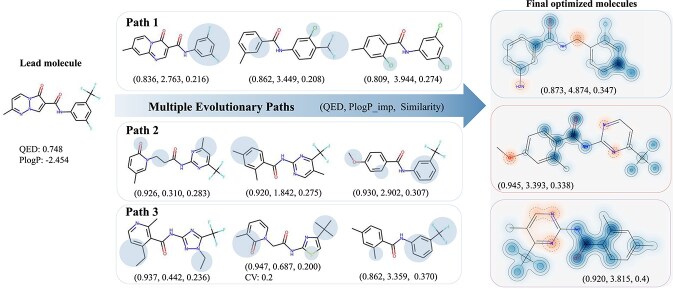
Multiple evolutionary paths formed by the optimized molecules of CMOMO for a lead molecule. The figure consists of three rows showing the evolutionary paths from the lead molecule to the final optimized molecules. The shaded regions in each row highlight the modified substructures. The three Tanimoto similarity maps in the last column show the similarity between the final optimized molecules and the lead molecule.

Figure 7 presents the evolution trajectories of molecules obtained by CMOMO for a randomly selected lead molecule, where three evolutionary paths are shown. The blue shaded regions in each path denote the modified substructures, the final optimized molecules are provided by plotting three Tanimoto similarity maps in the last column, where similar and dissimilar regions in comparison to the lead molecule are indicated by blue and red colors, respectively. From the figure, it can be seen that the lead molecule can evolve through diverse paths to obtain different successfully optimized molecules that make good trade-off between different properties. Besides, as shown in Path 3, some infeasible molecules that violate constraints can also be saved in the evolutionary process, which enables the lead molecule to be optimized through multiple evolutionary paths; thus, improving the exploration ability of the proposed CMOMO.

### Component effectiveness analysis

In this subsection, we conduct some ablation experiments to validate the effectiveness of key components in CMOMO by designing three algorithmic variants, namely CMOMO_nobank, CMOMO_noagg, and CMOMO_novfer. Specifically, the variant CMOMO_nobank is obtained by removing the Bank library from the population initialization of CMOMO, the variant CMOMO_noagg is obtained by replacing the ranking aggregation strategy in the constrained optimization stage of CMOMO with the constraint dominance principle [36], which is widely used as a constraint handling technique to address constraints, and the variant CMOMO_novfer is designed by replacing the VFER strategy in CMOMO with the polynomial mutation operation, which is widely used by many methods [26, 40] to generate the latent vectors of offspring molecules.

**Figure 8:**
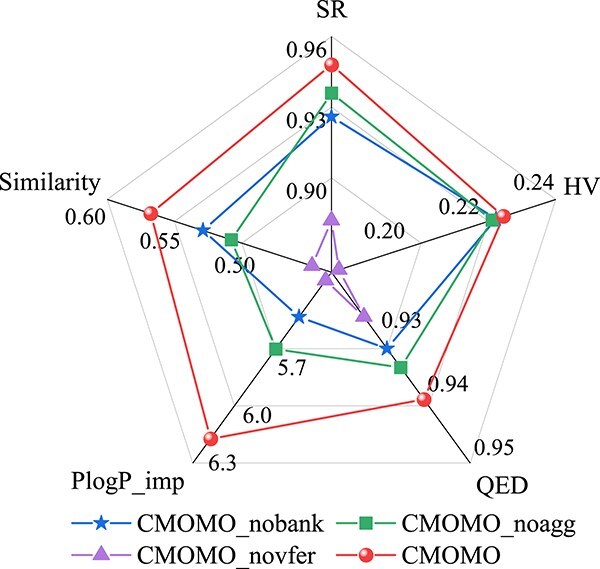
The performance of CMOMO and its three variants on Task 1.

Figure 8 provides the results of performance comparisons between CMOMO and its three variants on Task 1. It can be seen that CMOMO outperforms the three variants in terms of SR, HV, and three property values, including QED, PlogP_imp, and Similarity, which verifies that the three components in CMOMO are effective to obtain good performance. Moreover, it can be seen that the performance of CMOMO_novfer is obviously inferior to CMOMO when the VFER strategy is removed from CMOMO, which means that this evolutionary reproduction strategy has the most important effect on the performance of the proposed CMOMO. In high-dimensional multi-objective optimization problems, the traditional mutate strategies often fail to generate meaningful solutions, which can easily cause the optimization process to descend into local optima. In comparison, the fragmentation-based reproduction strategy splits the latent vector into several groups, and mutates each element in the group, which facilitates widely exploration of the space and escaping sub-optimal solutions.

Although the effects of the Bank library and the ranking aggregation strategy on the performance of CMOMO are not as obvious as that of the latent VFER strategy, their effectiveness are also not negligible. For example, when the Bank library is removed from the population initialization of CMOMO to obtain the variant CMOMO_nobank, the performance of CMOMO on QED and PlogP improvement metrics degrades considerably. The results show that the optimization process can be improved by using the Bank library to generate high-quality initial population. Besides, the ranking aggregation strategy in the constrained optimization stage adaptively balances property prioritization and constraint satisfaction, which utilizes infeasible high-property molecules to guide the exploration of the CPF.

## Conclusion

In this paper, we have proposed CMOMO, a deep constrained multi-objective optimization framework that can be readily adapted to any stringent drug-like criteria and any evaluation metrics of molecular property. It features an efficient dynamic cooperative optimization that enables CMOMO to balance property optimization and constraint satisfaction. The proposed CMOMO is able to simultaneously optimize multiple molecular properties while satisfying several stringent drug-like criteria. Further, the CMOMO framework can be applied to the tasks with other kinds of constraints, and other types of drugs, such as peptide, and proteins.

CMOMO demonstrates its superior performance over baseline results on the simpler benchmark tasks and two practical molecular optimization tasks, while adhering to two constraints. The CMOMO-optimized molecules of existing drug molecules show a favorable docking score with $\beta $2-adrenoceptor GPCR and drug likeness score, and satisfy constraints. Besides, the CMOMO-optimized molecules are consistently predicted to inhibit against glycogen synthase kinase-3$\beta $ and easily be synthesized by property predictors. Compared to the five competitors, the proposed CMOMO obtains more successfully optimized feasible molecules with better property values, which verifies the superiority of balancing property optimization and constraint satisfaction. The multiple evolutionary paths analysis provides insight into how CMOMO can efficiently explore the molecular space to discover a diverse set of improved molecules that possess the desired properties while adhering to established constraints. In addition, the effectiveness of the components used in CMOMO have been validated by component effectiveness analysis experiment. The results show strong evidence that CMOMO can serve as a novel and practical tool for molecule optimization to accelerate drug discovery with constraints.

**Key Points**

- One of the fundamental challenges of molecular optimization is how to simultaneously optimize multiple properties while satisfies several drug-like constraints. Few of previous molecule optimization methods pay attention to the treatment of constraints that are widely present in molecular optimization.
- To address the constrained multi-property molecular optimization problems, we developed a constrained multi-objective molecular optimization framework (CMOMO), which serves as a flexible and efficient method for optimizing multiple molecular properties simultaneously while satisfies several drug-like constraints.
- Compared with state-of-the-art methods, CMOMO facilitates simultaneous optimization of multiple molecular properties while adhering to various drug-like constraints, and subsequently identifies a set of optimal molecules with trade-offs among multiple objectives.

## Supplementary Material

CMOMO-SI-BIB_bbaf335

## Data Availability

The datasets used in this project are updated and available at https://github.com/ahu-bioinf-lab/CMOMO-master.

## Notes

The supplementary materials contains the details of experiment settings, the analysis of Bank library, invalid molecules, decay functions, run-times, and supplementary results.

A link to access the supplementary materials will be provided in the published article.

## Code availability

All of the codes are updated and available at https://github.com/ahu-bioinf-lab/CMOMO-master.

## References

[1] Sheng C, Li J. Structural Optimization of Drugs: Design Strategies and Empirical Rules, Vol. 7. Beijing: Chemical Industry Press, 2017.

[2] Nigam AK, Pollice R, Krenn M. et al. Beyond generative models: Superfast traversal, optimization, novelty, exploration and discovery (stoned) algorithm for molecules using selfies. *Chem Sci* 2021;12:7079–90. 10.1039/D1SC00231G 34123336 PMC8153210

[3] Fromer JC, Coley CW. Computer-aided multi-objective optimization in small molecule discovery. *Patterns* 2023;4:100678. 10.1016/j.patter.2023.100678 36873904 PMC9982302

[4] Li S, Cao L, Yang X. et al. Simultaneously optimizing multiple properties of $\beta $-glucosidase bgl6 using combined (semi-) rational design strategies and investigation of the underlying mechanisms. *Bioresour Technol* 2023;374:128792. 10.1016/j.biortech.2023.128792 36842511

[5] Lee Y, Choi K, Kim C. Docking-based multi-objective molecular optimization pipeline using structure-constrained genetic algorithm. In: 2022 IEEE International Conference on Bioinformatics and Biomedicine (BIBM), pp. 3438–45. IEEE, 2022.

[6] Wang J, Hsieh C-Y, Wang M. et al. Multi-constraint molecular generation based on conditional transformer, knowledge distillation and reinforcement learning. *Nature Machine Intelligence* 2021;3:914–22. 10.1038/s42256-021-00403-1

[7] Zhavoronkov A, Ivanenkov YA, Aliper A. et al. Deep learning enables rapid identification of potent ddr1 kinase inhibitors. *Nat Biotechnol* 2019;37:1038–40. 10.1038/s41587-019-0224-x 31477924

[8] Jin W, Barzilay R, Jaakkola T. Junction tree variational autoencoder for molecular graph generation. In: *International Conference on Machine Learning*, pp. 2323–32. PMLR, 2018.

[9] Liu Q, Allamanis M, Brockschmidt M. et al. Constrained graph variational autoencoders for molecule design. In: *Advances in Neural Information Processing Systems* 2018;31.

[10] Eckmann P, Sun K, Zhao B. et al. LIMO: latent inceptionism for targeted molecule generation. *Proceedings of Machine Learning Research* 2022;162:5777–92. 36193121 PMC9527083

[11] Xie Y, Shi C, Zhou H. et al. Markov molecular sampling for multi-objective drug discovery. In: Proceedings of the International Conference on Learning Representations. *Yong Yu, and lei Li*. Mars, 2021.

[12] Xia X, Zhang Y, Zeng X. et al. Artificial intelligence in molecular optimization: Current paradigms and future frontiers. *Int J Mol Sci* 2025;26:4878. 10.3390/ijms26104878 40430017 PMC12112088

[13] Nigam AK, Friederich P, Krenn M. et al. Augmenting genetic algorithms with deep neural networks for exploring the chemical space. *In ICLR* 2020.

[14] Ahn S, Kim J, Lee H. et al. Guiding deep molecular optimization with genetic exploration. *Advances in Neural Information Processing Systems* 2020;33:12008–21.

[15] Olivecrona M, Blaschke T, Engkvist O. et al. Molecular de-novo design through deep reinforcement learning. *J Chem* 2017;9:1–14. 10.1186/s13321-017-0235-x PMC5583141 29086083

[16] Jin W, Barzilay R, Jaakkola T. Multi-objective molecule generation using interpretable substructures. In: *International Conference on Machine Learning*, pp. 4849–59. PMLR, 2020.

[17] Gao K, Nguyen DD, Meihua T. et al. Generative network complex for the automated generation of drug-like molecules. *J Chem Inf Model* 2020;60:5682–98. 10.1021/acs.jcim.0c00599 32686938 PMC8142330

[18] Chen Z, Min MR, Parthasarathy S. et al. A deep generative model for molecule optimization via one fragment modification. *Nat Mach Intell* 2021;3:1040–9. 10.1038/s42256-021-00410-2 35187404 PMC8856604

[19] Zhang Y, Tong Y, Xia X. et al. A domain-label-guided translation model for molecular optimization. *Methods* 2024;224:71–8. 10.1016/j.ymeth.2024.02.005 38395182

[20] Sun M, Xing J, Meng H. et al. Molsearch: Search-based multi-objective molecular generation and property optimization. In: Proceedings of the 28th ACM SIGKDD Conference on Knowledge Discovery and Data Mining, pp. 4724–32, 2022. 10.1145/3534678.3542676 PMC10097503 37056719

[21] Jin W, Yang K, Barzilay R. et al. Learning Multimodal Graph-to-Graph Translation for Molecular Optimization. In: Proceedings of the International Conference on Learning Representations, 2019.

[22] Junchi Y, Tingyang X, Rong Y. et al. Structure-aware conditional variational auto-encoder for constrained molecule optimization. *Pattern Recognition* 2022;126:108581.

[23] Xia X, Zeng X, Zhang X. et al. Leveraging adaptive evolutionary optimization for drug molecular design involving many properties. *Big Data Min Anal* 2025. in press

[24] Hoffman SC, Chenthamarakshan V, Wadhawan K. et al. Optimizing molecules using efficient queries from property evaluations. *Nature*. *Mach Intell* 2022;4:21–31.

[25] Kwon Y, Lee J. Molfinder: an evolutionary algorithm for the global optimization of molecular properties and the extensive exploration of chemical space using smiles. *J Chem* 2021;13:1–14. 10.1186/s13321-021-00501-7 PMC7977239 33736687

[26] Xia X, Liu Y, Zheng C. et al. Evolutionary multiobjective molecule optimization in an implicit chemical space. *J Chem Inf Model* 2024;64:5161–74. 10.1021/acs.jcim.4c00031 38870455 PMC11235097

[27] Winter R, Montanari F, Steffen A. et al. Efficient multi-objective molecular optimization in a continuous latent space. *Chem Sci* 2019;10:8016–24. 10.1039/C9SC01928F 31853357 PMC6836962

[28] Verhellen J . Graph-based molecular pareto optimisation. *Chem Sci* 2022;13:7526–35. 10.1039/D2SC00821A 35872811 PMC9241971

[29] Zhang Y, Jiang H, Tian Y. et al. Multigranularity surrogate modeling for evolutionary multiobjective optimization with expensive constraints. *IEEE Trans Neural Networks Learn Syst* 2023;35:2956–68. 10.1109/TNNLS.2023.3297624 37527320

[30] Zhang Y, Tian Y, Jiang H. et al. Design and analysis of helper-problem-assisted evolutionary algorithm for constrained multiobjective optimization. *Inform Sci* 2023;648:119547. 10.1016/j.ins.2023.119547

[31] Thomas M, O’Boyle NM, Bender A. et al. Molscore: A scoring and evaluation framework for de novo drug design. 2024. 10.1186/s13321-024-00861-w PMC11141043 38816825

[32] Baell JB, Holloway GA. New substructure filters for removal of pan assay interference compounds (pains) from screening libraries and for their exclusion in bioassays. *J Med Chem* 2010;53:2719–40. 10.1021/jm901137j 20131845

[33] Larry Yet . Privileged Structures in Drug Discovery: Medicinal Chemistry and Synthesis. John Wiley & Sons, 2018, 10.1002/9781118686263.

[34] Winter R, Montanari F, Noé F. et al. Learning continuous and data-driven molecular descriptors by translating equivalent chemical representations. *Chem Sci* 2019;10:1692–701. 10.1039/C8SC04175J 30842833 PMC6368215

[35] Takahashi M, Kita H. A crossover operator using independent component analysis for real-coded genetic algorithms. In: Proceedings of the 2001 congress on evolutionary computation (IEEE Cat. No.01th8546), Vol. 1, pp. 643–9. IEEE, 2001.

[36] Deb K, Pratap A, Agarwal S. et al. A fast and elitist multiobjective genetic algorithm: NSGA-II. *IEEE Trans Evol Comput* 2002;6:182–97.

[37] Sterling T, Irwin JJ. ZINC 15–ligand discovery for everyone. *J Chem Inf Model* 2015;55:2324–37. 10.1021/acs.jcim.5b00559 26479676 PMC4658288

[38] Gaulton A, Bellis LJ, Patricia Bento A. et al. ChEMBL: a large-scale bioactivity database for drug discovery. *Nucleic Acids Res* 2012;40:D1100–7. 10.1093/nar/gkr777 21948594 PMC3245175

[39] Kim S, Thiessen PA, Bolton EE. et al. Pubchem substance and compound databases. *Nucleic Acids Res* 2016;44:D1202–13. 10.1093/nar/gkv951 26400175 PMC4702940

[40] Grantham K, Mukaidaisi M, Ooi HK. et al. Deep evolutionary learning for molecular design. *IEEE Comput Intell Mag* 2022;17:14–28. 10.1109/MCI.2022.3155308

[41] Richard Bickerton G, Paolini GV, Besnard J. et al. Quantifying the chemical beauty of drugs. *Nat Chem* 2012;4:90–8. 10.1038/nchem.1243 22270643 PMC3524573

[42] Daina A, Michielin O, Zoete V. iLOGP: a simple, robust, and efficient description of *n*-octanol/water partition coefficient for drug design using the GB/SA approach. *J Chem Inf Model* 2014;54:3284–301. 10.1021/ci500467k 25382374

[43] Bajusz D, Rácz A, Héberger K. Why is tanimoto index an appropriate choice for fingerprint-based similarity calculations? *J Chem* 2015;7:1–13. 10.1186/s13321-015-0069-3 PMC4456712 26052348

[44] Brown N, Fiscato M, Segler MHS. et al. GuacaMol: benchmarking models for de novo molecular design. *J Chem Inf Model* 2019;59:1096–108. 10.1021/acs.jcim.8b00839 30887799

[45] Nigam AK, Pollice R, Tom G. et al. Tartarus: a benchmarking platform for realistic and practical inverse molecular design. *Advances in Neural Information Processing Systems* 2023;36:3263–306.

[46] Eberhardt J, Santos-Martins D, Tillack AF. et al. Autodock vina 1.2. 0: new docking methods, expanded force field, and python bindings. *J Chem Inf Model* 2021;61:3891–8. 10.1021/acs.jcim.1c00203 34278794 PMC10683950

[47] Kaminsky N, Singer U, Radinsky K. CFOM: lead optimization for drug discovery with limited data. In: Proceedings of the 32nd ACM International Conference on Information and Knowledge Management, pp. 1056–66, 2023.

[48] Li Y, Zhang L, Liu Z. Multi-objective de novo drug design with conditional graph generative model. *J Chem* 2018;10:1–24. 10.1186/s13321-018-0287-6 PMC6057868 30043127

[49] Huang K, Tianfan F, Gao W. et al. Therapeutics data commons: machine learning datasets and tasks for drug discovery and development. *Advances in Neural Information Processing Systems* 2021.

[50] Jensen JH . A graph-based genetic algorithm and generative model/Monte Carlo tree search for the exploration of chemical space. *Chem Sci* 2019;10:3567–72. 10.1039/c8sc05372c 30996948 PMC6438151

[51] While L, Hingston P, Barone L. et al. A faster algorithm for calculating hypervolume. *IEEE Trans Evol Comput* 2006;10:29–38. 10.1109/TEVC.2005.851275

[52] Kagami LP, das GM, LFSM. et al. Geo-measures: a PyMOL plugin for protein structure ensembles analysis. *Comput Biol Chem* 2020;87:107322. 10.1016/j.compbiolchem.2020.107322 32604028

[53] Li H, Zhang R, Min Y. et al. A knowledge-guided pre-training framework for improving molecular representation learning. *Nat Commun* 2023;14:7568. 10.1038/s41467-023-43214-1 37989998 PMC10663446

[54] Ahmad S, Waheed Y, Abro A. et al. Molecular screening of glycyrrhizin-based inhibitors against ACE2 host receptor of SARS-CoV-2. *J Mol Model* 2021;27:206. 34169390 10.1007/s00894-021-04816-y PMC8225399

[55] UniProt Consortium . Uniprot: A worldwide hub of protein knowledge. *Nucleic Acids Res* 2019;47:D506–15. 10.1093/nar/gky1049 30395287 PMC6323992

