# Supplementary material for: CMOMO: a deep multi-objective optimization framework for constrained molecular multi-property optimization
Source: Brief Bioinform. 2025 Jul 10;26(4):bbaf335. doi: 10.1093/bib/bbaf335 (PMC12240737; doi:10.1093/bib/bbaf335)
Supplement: CMOMO-SI-BIB_bbaf335 [file cmomo-si-bib_bbaf335.docx]

**Supplementary Information:**

**CMOMO: a deep multi-objective optimization framework for constrained molecular multi-property optimization**

Xin Xia^1^, Yajie Zhang^2^, Xiangxiang Zeng^3^, Xingyi Zhang^2^, Chunhou Zheng^1^, and Yansen Su^1^*

Email: [suyansen@ahu.edu.cn](mailto:suyansen@ahu.edu.cn,)

**The Supplementary Materials file includes：**

**S1. Analysis of the generated invalid molecules**

**S2. Population initialization**

**S3. NSGA-II algorithm**

**S4. Analysis of different decay functions in CMOMO**

**S5. Experiment settings**

**S6. Analysis of Bank library**

**S7. Property values of optimized molecules**

**S8. Number of successfully optimized molecules**

**S9. Significance analysis on four tasks**

**S10. Comparisons of run-times**

**S11. Analysis of successfully optimized molecules**

**S12. References**

**S1. Analysis of the generated invalid molecules**

To evaluate the proportion of invalid molecules generated by different methods, we conducted experiments on the Task1 dataset using 100 randomly selected lead molecules with a population size of 100 and 100 generations. Then, we calculated the ratio of invalid SMILES (number of invalid molecules/total generated molecules) during the optimization of each lead molecule. As shown in **Table S1**, QMO, MOMO, and CMOMO all demonstrate a low rate of invalid molecule generation per lead molecule (below 1%). Among all methods, MolFinder, which employs molecular sequence-based evolution, exhibits the highest proportion of invalid molecules. This stems from its reliance on SMILES sequence crossover and atomic insertion/deletion operations, which frequently produce invalid SMILES. GB-GA-P, which is based on discrete molecular graph evolution, also exhibits a relatively high rate of invalid molecules.

**Table. S1 The validity ratio of invalid molecules against total generated molecules across the six methods on Task 1.**

|  | **Ratio of invalid molecules** |
| --- | --- |
| **QMO** | 0.48% |
| **Molfinder** | 87.76% |
| **MOMO** | 0.92% |
| **MSO** | 1.47% |
| **GB-GA-P** | 23.07% |
| **CMOMO** | 0.97% |

These statistical results demonstrate that evolution in the latent space using a pre-trained encoder-decoder architecture significantly enhances the generation of chemically valid molecular structures. The small ratio of invalid molecules (below 1%) generated during the evolution only marginally increases the computational overhead and has a negligible effect on the optimization results.

**S2: Population initialization**

For a given lead molecule, the high-property molecules in the public database that are similar to the lead molecule are selected for Bank (**Fig. S1A**). The lead molecule and Bank molecules are encoded into latent vectors, and new vectors in initial population are generated by crossover (**Fig. S1B**).


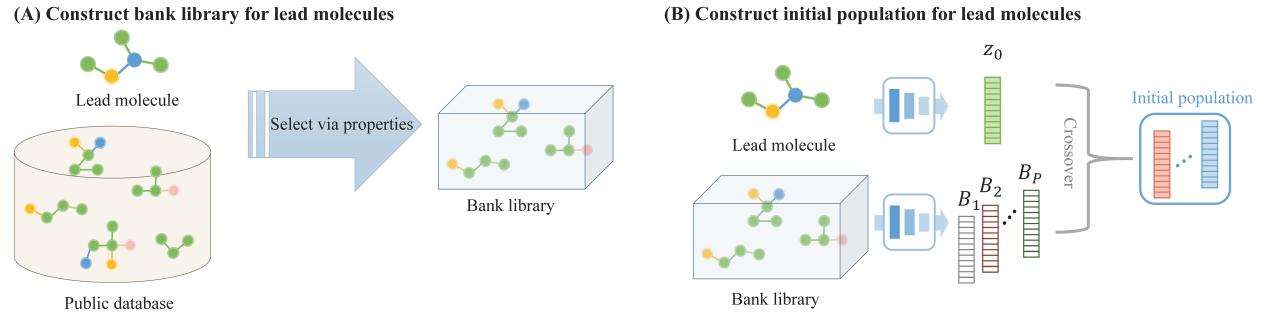


**Fig. S1 The procedure to generate the Bank library and initial population for a lead molecule.**

**S3: NSGA-II algorithm**

**Non-dominated sorting.** The non-dominated sorting is used to assign molecules to different fronts according to their dominance relationship, which facilitates to improve the convergence of molecular population by selecting molecules with smaller front number. Typically, molecules with smaller front number are the ones with good convergence. Herein, we first introduce how to determine the dominance relationship between different molecules. Given two molecules $x_{1}$ and $x_{2}$, the molecule $x_{1}$ is said to dominate molecule $x_{2}$ (denoted as $x_{1}\prec x_{2}$) if and only if $x_{1}$ is not worse than $x_{2}$ on any objective, besides, the molecule $x_{1}$ is better than $x_{2}$ on at least one objective. Given that the collection of all molecules to be sorted is termed as $Q$, if there does not exist a molecule in $Q$ that dominates molecule $x_{1}$, the molecule $x_{1}$ is called a non-dominated molecule; otherwise, it is called a dominated molecule. In non-dominated sorting, all non-dominated molecules are first selected and removed from $Q$ to obtain the first front $F_{1}$. Then, the dominance relationship between the remaining molecules in $Q$ is recalculated with all non-dominated molecules being removed from $Q$ to obtain the second front $F_{2}$. The above operations repeat until all molecules in $Q$ are assigned to different fronts.

**Crowding distance calculation.** In the cast that the non-dominated sorting is used to improve the convergence of molecular population, molecules with the same front number are further sorted by their crowding distance, which helps to maintain the diversity of molecular population. Specifically, given a molecule $x$, its crowding distance is calculated as follows.

$CD(x)=\sum_{i=0}^{m} \frac{f_{i}(x_{a})-f_{i}(x_{b})}{f_{i}^{max}-f_{i}^{min}}$*,*

where $f_{i}^{max}$ and $f_{i}^{min}$ are the maximum and minimum objective values of all molecules on the $i$-th objective, $x_{a}$ and $x_{b}$ are two nearest neighborhood molecules to molecule $x$ with their objective values $f_{i}(x_{a})$ and $f_{i}(x_{b})$ being larger and smaller than $f_{i}(x)$, respectively.

**S4: Analysis of different decay functions in CMOMO**

we conduct experiments on 100 randomly selected lead molecules in Task 1 to compare the effects of different decay curves on the CMOMO. The CMOMO_linear variant employs a linear decay function defined as $\alpha=1-\frac{t}{T}$. The CMOMO_exponential variant employs an exponential decay function defined as $\alpha(t)=e^{\frac{ln(0.001)}{T}t}$. The CMOMO_cosine version, which utilizes the cosine decay function as presented in the manuscript, is defined as $\alpha(t)=\frac{1}{2}\times(1+cos(\frac{t}{T}\pi))$.

**Table S2: Performance of CMOMO with different decay functions on Task 1.**

|  | **SR** | **HV** | **Number_sr** |
| --- | --- | --- | --- |
| **QMO** | 0.39 | 0.149 | 1.412 |
| **Molfinder** | 0.31 | 0.235 | 2.580 |
| **MOMO** | 0.64 | 0.239 | 4.953 |
| **MSO** | 0.53 | 0.238 | 6.840 |
| **GB-GA-P** | 0.53 | 0.297 | 5.830 |
| **CMOMO_linear** | **0.94** | 0.326 | 15.05 |
| **CMOMO_exponential** | 0.93 | 0.324 | 15.53 |
| **CMOMO_cosine** | **0.94** | **0.329** | **15.74** |

**Table S2** presents a comparative evaluation of five baseline methods and three variants (CMOMO_linear, CMOMO_cosine, CMOMO_exponential) on Task 1, with performance metrics including success rate (SR), average hypervolume (HV), and mean number of successfully optimized molecules per lead compound (Number_sr). The results demonstrate that all three CMOMO variants with distinct decay functions significantly outperform the five baseline methods, indicating that various decay formulations can effectively enhance the optimization capability of CMOMO.


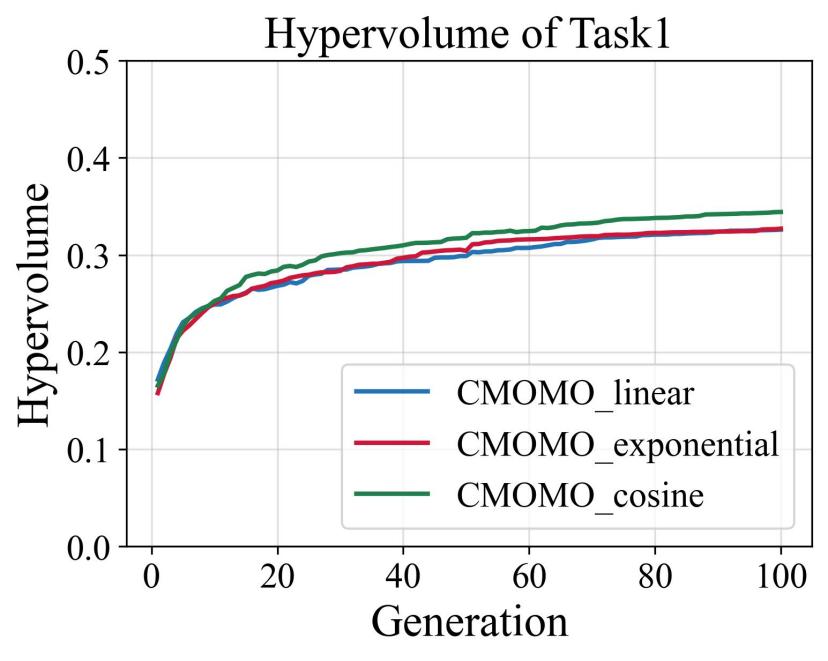


**Fig. S2 The property values of molecules optimized by CMOMO and comparison methods on four tasks.**

Moreover, using the hypervolume (HV) as an example, we illustrate the hypervolume curves of the three variants in **Fig. S2** throughout the optimization iterations. The results demonstrate that the cosine-based CMOMO maintains relatively higher HV values compared to the other variants.

**S5. Experiment settings**

**S5.1 Bank library**

The detailed bank selection procedure consists of three steps for each lead molecule. First, we calculate the target property values and Tanimoto similarity scores for all candidate molecules in public databases. Then, we define thresholds for target properties to select molecules with properties larger than the thresholds. The thresholds are set based on the number of molecules that are better than the thresholds. Finally, we select the top B molecules with the largest Tanimoto similarity with the lead molecule to form the bank library (where B denotes the Bank size). The specific selection criteria, Bank sizes, and data sources for the four tasks are set as follows.

**Task 1.** We screen 200 molecules from ZINC^1^ to construct a Bank library for each lead molecule. The screened molecules are the 200 molecules most similar to the lead molecule with QED larger than 0.8 and PlogP larger than 2.

**Task 2.** We screen 200 molecules from the dataset provided by Brown et al.^2^ to construct a Bank library for each lead molecule. The screened molecules are the 200 molecules most similar to the lead molecule with mean values of three structural properties of molecules larger than 0.25.

**Task 3.** We screen 200 molecules from the publicly available dataset from Nigam et al.^3^ to construct the Bank library for each lead molecule. The screened molecules are most 200 molecules similar to the lead molecule with QED larger than 0.7 and 4LDE score smaller than -9.

**Task 4.** We screen 200 molecules from the dataset^4^ to construct the Bank library for each lead molecule. The screened molecules are most 200 molecules similar to the lead molecule with QED larger than 0.6, inhibition equal to 1, and SA larger than 0.5.

**S5.2 Parameter setting**

In CMOMO, the parameter $d=0.25$ used to control crossover (Equation 5 in Methods), the crossover probability $p_{c}=1$, the 512-dimensional vectors are divided into 16 small fragments in the fragmentation-based mutation, the mutation probability $p_{m}=0.5$, these parameters were consistent across all tasks. The population size and the number of iterations were adjusted based on the difficulty of the task and the running time. The optimization stages 1 and 2 are set with the same number of iterations in CMOMO, which is half of the total number of iterations. To ensure a fair comparison, the comparison model were configured with the same population size (or number of samples) and the same number of iterations for evolution or search. Population size/number of samples was 100, iterations was 100 (both stages 1 and 2 were 50 in CMOMO) on Task 1, Task 2, and Task4. Population size/number of samples = 50, iterations/evolution = 50 (both stages 1 and 2 were 25 in CMOMO) on Task 3. The objective function of QMO and MSO was the aggregation of all properties on four tasks.

**S5.3 Success thresholds for the four tasks.**

**Table S3: The success thresholds of CMOMO on four tasks.**

| **Task1** | **Task2** | **Task3** | **Task4** |
| --- | --- | --- | --- |
| QED $\geq0.85$  PlogP_imp $\geq3$  Similarity $\geq0.3$ | Score_dissim $\geq0.5$  Score_mw $\geq0.5$  Score_rb $\geq0.5$  Similarity $\geq0.3$ | QED $\geq0.8$  4LDE $\leq-10$  Similarity $\geq0.3$ | QED $\geq0.7$  GSK3$\beta$ inhibition $\geq0.4$  SA $\geq0.7$  Similarity$\geq0.2$ |

**S6. Analysis of Bank library**

To evaluate the impact of Bank size, we tested the performance of CMOMO with varying Bank sizes (B = 50, 100, 200, 300, 400, 500) on Task 1. The experiments were conducted using 20 randomly selected lead molecules from Task 1, with a population size of 100 and 100 evolutionary generations. **Fig. S3** illustrates the progression of population hypervolume (HV) across generations for different Bank sizes. The results demonstrate no significant differences in HV convergence or variation among the different Bank sizes. We attribute this observation to the fact that the Bank is solely utilized for crossover operations with lead molecules during initial population generation, its size does not affect the population size.

**
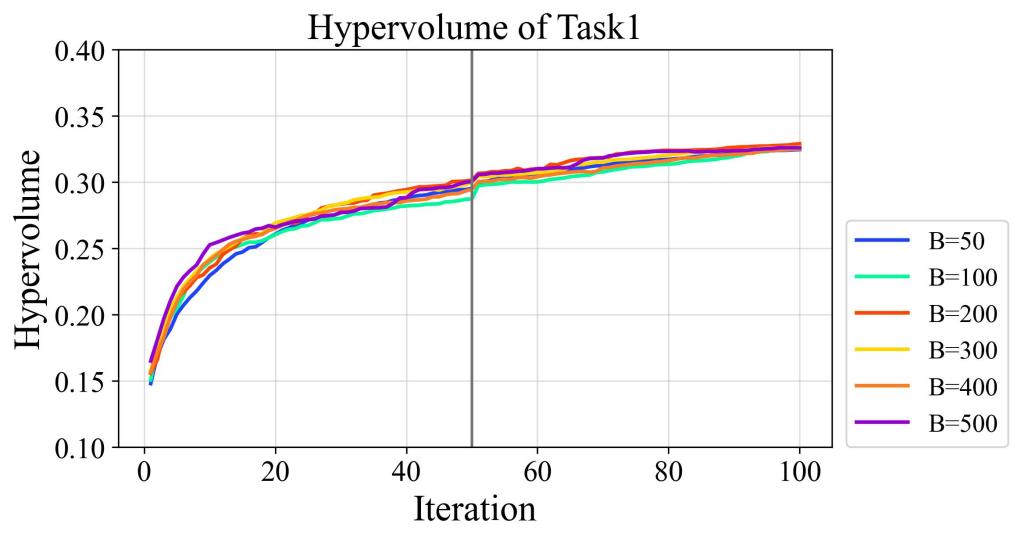
**

**Fig. S3. The curves of hypervolume of population with the iterative process under different Bank sizes on Task 1.**

Furthermore, **Fig. S4** presents the property distributions of successfully optimized molecules obtained with different Bank sizes, which shows that no discernible difference in the quality of optimized molecules. We set the size of the bank to 200 for the following reason. In the initial population generation, the generated molecules are selected through an elite selection strategy, which typically retains half of the molecules for the population. Thus, with a population size of 100, we set the bank size to 200 to generate 200 molecules for selection.

**
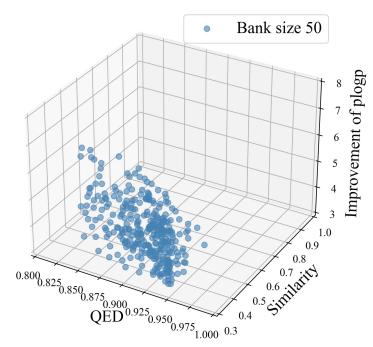

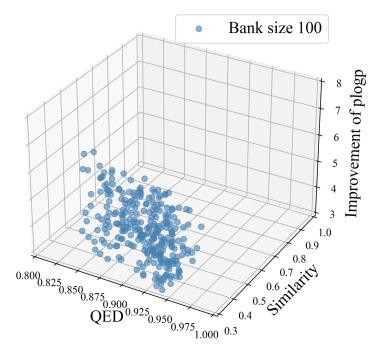

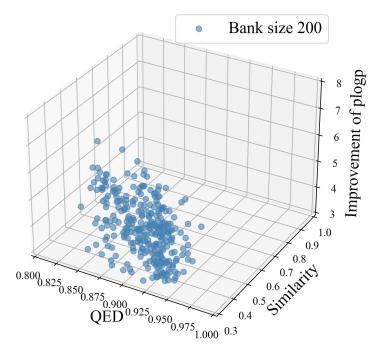
**

**
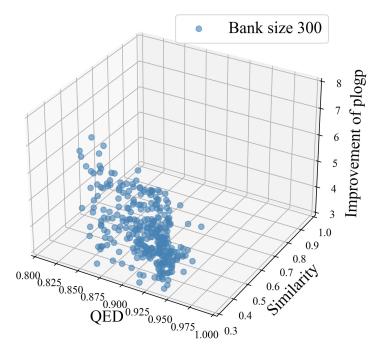

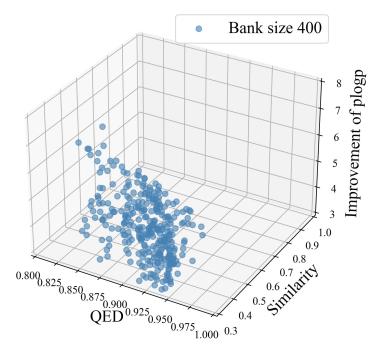

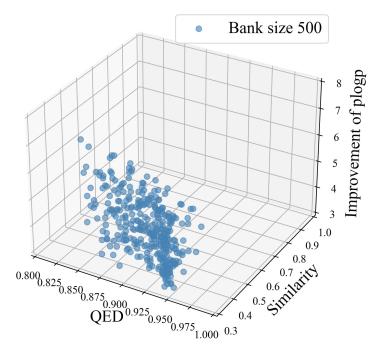
**

**Fig. S4. Properties of successfully optimized molecules obtained by CMOMO with different bank sizes on Task 1.**

**S7: Property values of optimized molecules**

**Fig. S5** presents the mean and standard deviation property values of molecules optimized by CMOMO and comparison methods on four tasks. Fig. S5A shows the QED, PlogP_imp and similarity of successful molecules optimized by CMOMO and comparison methods on Task 1. Fig. S5B shows the Score_dissim, Score_mw, Score_rb and similarity of successfully optimized molecules on Task 2. Fig. S5C presents QED, absolute value of 4LDE and similarity of successfully optimized molecules on Task 3. The QED, GSK3b, SA and similarity of successfully optimized molecules on Task 4 are drawn in Fig. S5D.


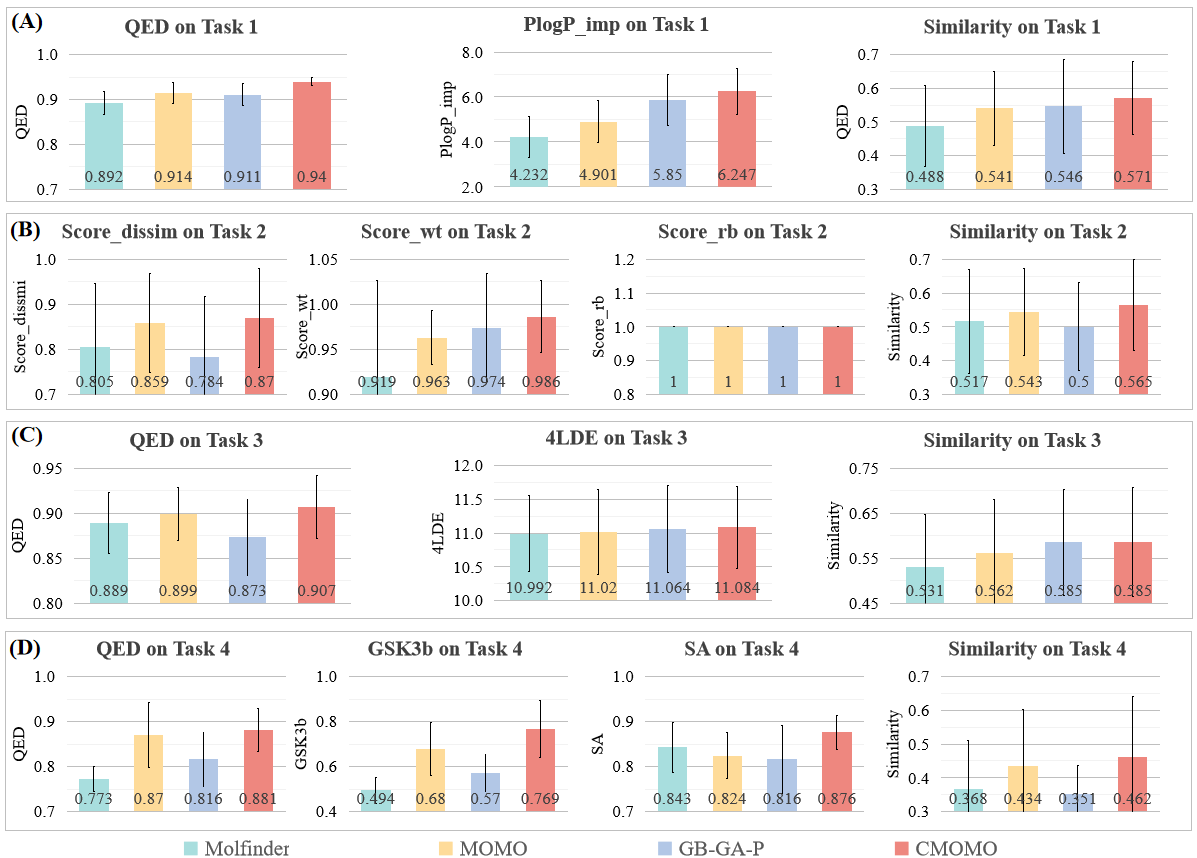


**Fig. S5. The mean and standard deviation property values of molecules optimized by CMOMO and comparison methods on four tasks.**

**S8: Number of successfully optimized molecules**

Number of successfully optimized molecules per lead molecule for CMOMO and three compared multi-objective based optimization methods on four tasks are shown in **Fig. S6**. Each row corresponds to the four optimization methods, and each column corresponds to the lead molecules in the dataset. The darker the color, the more successful molecules are optimized. (A) The number of successfully optimized molecules on Task 1. (B) The number of successfully optimized molecules on Task 2. (C) The number of successfully optimized molecules on Task 3. (D) The number of successfully optimized molecules on Task 4.


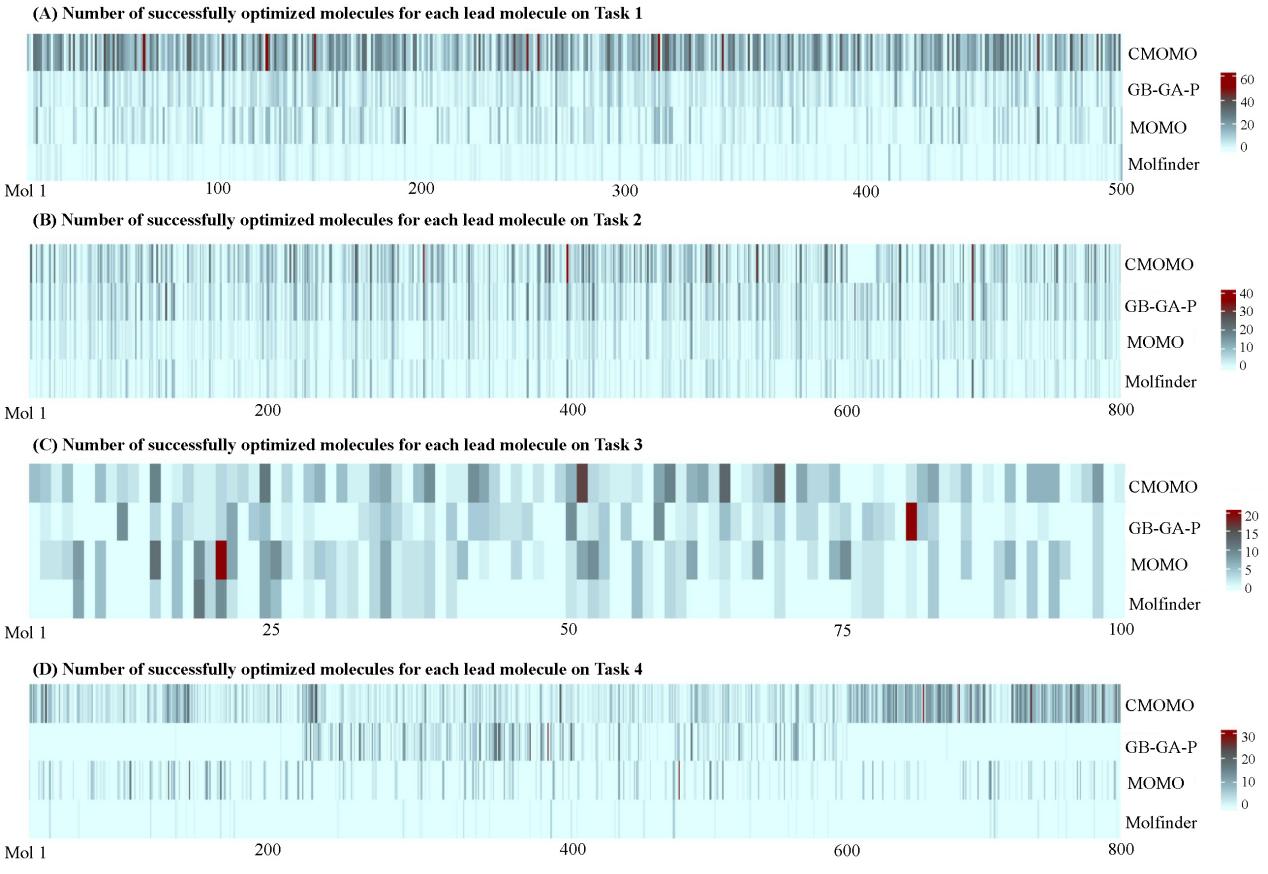


**Fig. S6. Number of successfully optimized molecules per lead molecule for CMOMO and three compared multi-objective based optimization methods on four tasks.**

**S9: Significance analysis on four tasks**

To validate the reliability of the optimization results, a significance analysis is conducted. Specifically, the Wilcoxon signed-rank test is applied to compare the optimization results of compared methods with CMOMO across lead molecules, thereby verifying whether the results of CMOMO are significantly superior to those of the compared methods. The number of successfully optimized molecules (Number_sr) and the hypervolume (HV) of six methods on four tasks are analyzed for significance.

The assumption $H_{0}$ is that the median difference between CMOMO's results and the baseline methods' results is less than or equal to zero, indicating CMOMO does not outperform the baseline methods. The alternative hypothesis $H_{1}$ is that the median difference is larger than zero, i.e., CMOMO significantly outperforms the baseline methods. If the p-value is less than 0.05, the assumption $H_{0}$ is rejected, and the assumption $H_{1}$ is accepted, i.e., the results of CMOMO are significantly better than those of the baseline methods.

**Table S4-S7** presents the p-values of CMOMO compared to each of the five methods on Task 1 to Task 4, respectively. It is evident that the p-values for both SR and HV on the four tasks are smaller than 0.05, demonstrating that CMOMO significantly outperforms the comparison methods.

**Table S4: Statistical sianificance test of performance of CMOMO and comparison methods on Task 1.**

|  | **P value of Number_sr** | **P value of HV** |
| --- | --- | --- |
| **CMOMO & QMO** | $1.652\times{10}^{-79}$ | $6.323\times{10}^{-84}$ |
| **CMOMO & Molfinder** | $2.487\times{10}^{-79}$ | $3.922\times{10}^{-75}$ |
| **CMOMO & MOMO** | $1.131\times{10}^{-75}$ | $5.321\times{10}^{-63}$ |
| **CMOMO & MSO** | $9.457\times{10}^{-56}$ | $2.501\times{10}^{-70}$ |
| **CMOMO & GB-GA-P** | $1.503\times{10}^{-74}$ | $9.271\times{10}^{-15}$ |

**Table S5: Statistical sianificance test of performance of CMOMO and comparison methods on Task 2.**

|  | **P value of Number_sr** | **P value of HV** |
| --- | --- | --- |
| **CMOMO & QMO** | $5.027\times{10}^{-106}$ | $6.994\times{10}^{-127}$ |
| **CMOMO & Molfinder** | $2.986\times{10}^{-81}$ | $3.410\times{10}^{-118}$ |
| **CMOMO & MOMO** | $1.071\times{10}^{-26}$ | $3.335\times{10}^{-79}$ |
| **CMOMO & MSO** | $3.474\times{10}^{-102}$ | $2.378\times{10}^{-121}$ |
| **CMOMO & GB-GA-P** | $5.620\times{10}^{-66}$ | $3.379\times{10}^{-75}$ |

**Table S6: Statistical sianificance test of performance of CMOMO and comparison methods on Task 3.**

|  | **P value of Number_sr** | **P value of HV** |
| --- | --- | --- |
| **CMOMO & QMO** | $2.129\times{10}^{-14}$ | $1.768\times{10}^{-15}$ |
| **CMOMO & Molfinder** | $7.398\times{10}^{-7}$ | $1.345\times{10}^{-5}$ |
| **CMOMO & MOMO** | $0.123\times{10}^{-1}$ | $0.118\times{10}^{-1}$ |
| **CMOMO & MSO** | $6.875\times{10}^{-14}$ | $0.914\times{10}^{-2}$ |
| **CMOMO & GB-GA-P** | $6.695\times{10}^{-5}$ | $0.324\times{10}^{-1}$ |

**Table S7: Statistical sianificance test of performance of CMOMO and comparison methods on Task 4.**

|  | **P value of Number_sr** | **P value of HV** |
| --- | --- | --- |
| **CMOMO & QMO** | $1.616\times{10}^{-44}$ | $2.057\times{10}^{-118}$ |
| **CMOMO & Molfinder** | $1.940\times{10}^{-100}$ | $1.884\times{10}^{-117}$ |
| **CMOMO & MOMO** | $5.333\times{10}^{-38}$ | $8.392\times{10}^{-6}$ |
| **CMOMO & MSO** | $3.508\times{10}^{-42}$ | $7.864\times{10}^{-86}$ |
| **CMOMO & GB-GA-P** | $1.803\times{10}^{-57}$ | $5.008\times{10}^{-51}$ |

**S10. Comparisons of run-times**

We conducted a comparison of the average wall-clock times between CMOMO and baseline methods on Task 1. All experiments were performed under the same conditions, including equivalent population sizes/sampling numbers and iterations. **Table S8** presents the comparative analysis, detailing the mean runtime (in minutes) and variance for each method.

**Table S8: Runtimes of different methods on Task 1 (Unit: minutes).**

|  | **P value of Number_sr** |
| --- | --- |
| **QMO** | 29.23 ± 3.027 |
| **Molfinder** | 2.070 ± 0.620 |
| **MOMO** | 33.32 ± 4.463 |
| **MSO** | 87.00 ± 33.51 |
| **GB-GA-P** | 10.33 ± 6.710 |
| **CMOMO** | 55.28 ± 3.430 |

As shown in **Table S8**, the continuous space-based methods (QMO, MOMO, MSO, CMOMO) exhibit significantly longer average runtime (29-87 minutes) compared to the discrete space methods (MolFinder: 2 minutes, GB-GA: 10 minutes) due to their iterative molecular encoding-decoding procedure. Among these methods, MSO requires the most computational time (about 87 minutes). CMOMO is computationally more expensive than the discard-based methods (QMO and MOMO) due to the repeated constraint evaluations in its dynamic constraint handling strategy. Importantly, CMOMO demonstrates superior performance in constrained multi-property optimization tasks (see Figures 4-6 in the main text). For example, in Task 1, CMOMO achieves a success rate (SR) of 94.8%, representing a 26.4% improvement over the best baseline method (68.4%). To address the computational cost, we plan to implement lightweight surrogate models in the latent space to replace the time-consuming encoding-decoding step in future work, thereby improving the optimization efficiency.

**S11. Analysis of successfully optimized molecules**

**
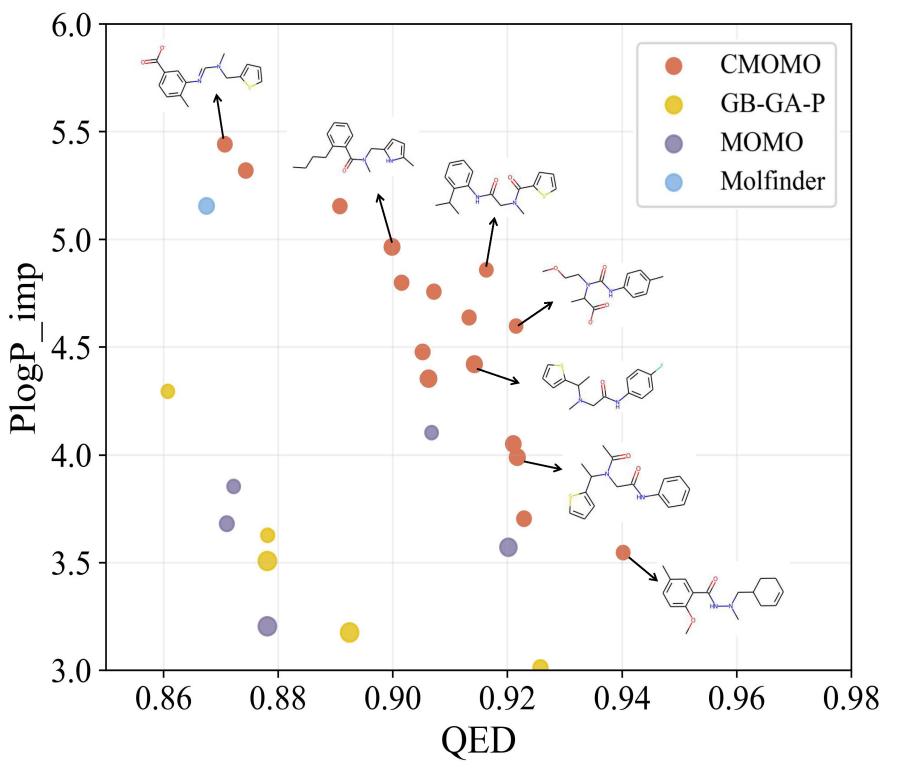
**

**Fig. S7. Comparison of successfully optimized molecules obtained by CMOMO and three Pareto optimization methods for a lead molecule.**

**Fig. S7** shows the properties of successfully optimized molecules obtained by CMOMO and three Pareto optimization methods for a lead molecule. The structures of some molecules obtained by CMOMO are displayed next to the corresponding dots.

**S12. References**

1. Sterling, T.; Irwin, J. J. ZINC 15-ligand discovery for everyone. J. Chem. Inf. Model. 55, 2324-2337 (2015).
2. Brown, N., Fiscato, M., Segler, M. H., Vaucher, A. C. GuacaMol: benchmarking models for de novo molecular design. J. Chem. Inf. Model. 59(3), 1096-1108. (2019).
3. Nigam, A., Pollice, R., Tom, G., Jorner, K., Thiede, L. A., Kundaje, A., Aspuru-Guzik, A. Tartarus: A benchmarking platform for realistic and practical inverse molecular design. arXiv preprint arXiv:2209.12487. (2022).
4. Jin W, Barzilay R, Jaakkola T. Multi-objective molecule generation using interpretable substructures. International conference on machine learning. PMLR, 4849-4859 (2020).
